# Preferences for fat, sugar, and oral-sensory food qualities in monkeys and humans

**DOI:** 10.1016/j.physbeh.2025.114998

**Published:** 2025-06-14

**Authors:** Fei-Yang Huang, Fabian Grabenhorst

**Affiliations:** Department of Experimental Psychology, University of Oxford, Mansfield Road, Oxford OX1 3TA, United Kingdom

**Keywords:** Nutrients, Oral texture, Nonhuman primates, Food choice, Reinforcement learning, Obesity, Dieting

## Abstract

In humans and other primates, food intake depends on sophisticated, individualized preferences for nutrients and oral-sensory food qualities that guide decision-making and eating behavior. The neural and behavioral mechanisms for such primate-typical food preferences remain poorly understood, despite their importance for human health and their targeting by pharmacological obesity treatments. Here, we review a series of experiments that investigated how the biologically critical properties of foods—their nutrients (sugar, fat, protein) and oral-sensory qualities (viscosity, oral sliding friction)—influence food preferences in monkeys and humans. In an economic nutrient-choice paradigm, macaques flexibly trade nutrients and oral-sensory food qualities against varying food amounts, consistent with the assignment of subjective values. Nutrient-value functions that link objective nutrient content to subjective values accurately model these preferences, predict choices across contexts, and explain individual differences. The monkeys’ aggregated choice patterns resulting from their nutrient preferences lead to daily nutrient balances that deviate from dietary reference points, resembling suboptimal human eating patterns when exposed to high-calorie foods. To investigate the sensory basis underlying nutrient values, we developed novel engineering tools that quantify food textures on oral surfaces, using fresh pig tongues. Oral-texture (i.e., mouthfeel) parameters, including viscosity and sliding friction, were shown to mediate monkeys’ preferences for high-fat foods. When translated to human subjects, this approach revealed a neural mechanism for preferring high-fat foods from oral texture in the orbitofrontal cortex (OFC)—a key reward system of the brain. Importantly, human OFC responses to oral sliding friction in individual subjects—measured in the MRI scanner—predicted subsequent fat intake in a naturalistic, life-like eating test. These findings suggest that a primate nutrient-reward paradigm offers a promising approach for investigating the behavioral and neural mechanisms for human-typical food reward and food choice, to advance understanding of human eating behavior, overeating, and obesity.

## 1 Introduction

The regulation of nutritional state and energy balance through controlled food intake is critical for health, well-being, and the prevention of obesity [1,2]. In primates including humans, food intake depends on sophisticated, highly individualized valuations of visual and oral-sensory food cues that guide learning, decision-making, and eating behavior [1,3–10]. Thus, in primates, “choosing what to eat” is a complex process that directly impacts nutritional health [11]. Although different macronutrients (fat, sugar, and protein) all serve as sources of energy, they each fulfil specific physiological functions and are thus not fully interchangeable. Accordingly, effective regulation of food intake to meet nutrient requirements must occur at the decision-making stage before food consumption [12]. To obtain specific nutrients, humans and animals rely on nutrient-specific sensory properties to identify these nutrients and adjust their food preferences [3,13,14]. Therefore, the neural mechanisms implementing food reward and choice must be sensitive to the nutrient content of available food options and related visual and oral-sensory cues. This view is supported by evidence that links the nutrient composition of foods to human sensory, hedonic, and economic valuations [14–17], and the activation of neural reward systems [4,8,18–25].

Food choice can be conceptualized as a decision problem based on ‘subjective value’—a foundational concept in behavioral theories that formalize learning and decision-making [26–28]. In economic theory, subjective value is inferred from observable choices and decision-making is modelled as an optimization process aimed at maximizing this value (‘utility’) [29,30]. In reinforcement learning (RL), subjective values of choice options are iteratively updated through trial-and-error experiences to guide future behavior [31]. Although these frameworks have been instrumental in identifying the behavioral and neural mechanisms underlying food choices, they cannot fully explain how food preferences are shaped by specific intrinsic properties of foods [32,33]. For example, while preference for sugar is nearly universal, there is substantial variability in individual preferences for fat [34]. Indeed, sensory scientists and food engineers study the principles that link food composition to palatability [3,13–15,17]. Investigating how neural reward systems construct food preferences by assigning value to biologically essential nutrients and related sensory food qualities can thus provide important mechanistic insights [2,6]. Notably, the behavioral and neural mechanisms for food choice constitute a prime target for interventions aiming to treat obesity and enhance nutritional health by modifying food preferences [1,35].

Unlike canonical economic and reinforcement learning theories, which do not conceptualize the origins of value in specific reward components, biology views choice objects as rewards endowed with intrinsic value derived from well-defined components that are essential for survival and reproductive success [36]. Here, we review a series of studies [4,6,37,38] that developed an approach to primate food choice based on nutrient reward and examined how the biologically critical properties of foods—their nutrients and sensory qualities—influence food preferences in macaques and humans.

## 2 A translational approach to nutrient reward in macaques and humans

Macaques, like humans, exhibit advanced capabilities in reward evaluation, allowing them to make sophisticated, value-based decisions [27,39–42]. In their natural habitats, macaques face diverse food sources with varying nutrient profiles, necessitating intricate decision-making to maintain nutritional balance [10]. Furthermore, the dynamic nature of their feeding environment requires macaques to adapt to both short-term and seasonal fluctuations in nutrient availability [43,44]. These features of macaques’ adaptive food decision-making resemble human food choice, particularly in how food rewards are evaluated and how choices are adjusted to meet fluctuating nutritional demands. Importantly, different from rodents, macaques and humans primarily rely on visual cues to identify food items; such visual food cues acquire value through associative learning with the food’s nutrient and oral-sensory components. Thus, nonhuman primates are especially suitable for studying human-typical decision-making processes guided by visual cues [5,45,46].

Given the rich behavioral and cognitive repertoire of macaques, their shared sensory and neural reward systems with humans, and their suitability for undergoing single-neuron recordings during behavioral tasks [44,47–53], macaques represent a valuable animal model for investigating neural reward processing underlying human-like feeding behavior and obesity [2,6,37,54].

The translational approach to primate nutrient reward reviewed in this paper [4,6,37] combines the careful design of food rewards in terms of their nutrients (fat, sugar, protein) and oral-sensory properties (viscosity, sliding friction) with well-controlled behavioral tasks designed to measure subjective food valuations (economic choices and reinforcement learning in macaques, psychophysics and economic auctions in humans). This nutrient-reward paradigm can serve as a tool to study the behavioral and neural mechanisms underlying primate-typical food intake, to complement genetic [55] and neural-circuit approaches [1,56,57] in other species, including approaches investigating gut-brain pathways underlying food reward [57].

## 3 Nutrients and oral-sensory food qualities as biological sources of economic values underlying food preferences

In a series of experiments, we aimed to empirically ground the concept of subjective value in the constitutive (nutrient and sensory) properties of food rewards using well-controlled repeated-choice paradigms from behavioral neurophysiology. Previous studies in macaques identified important factors influencing reward values, choices, and their neural mechanisms, including the assignment of values to choice options [40,42,58–64], reinforcement learning [27,65] and effects of satiety and thirst [3,66,67]. However, the influence of specific nutrients on formally defined reward values in macaques had remained unclear. We therefore manipulated the fat and sugar content of liquid food rewards that could be associated with visual conditioned stimuli and delivered in precise quantities. Using these stimuli, we tested whether the monkeys’ choices were sensitive to the nutrient composition of rewards, and reflected subjective trade-offs between nutrient composition and offered reward amounts, consistent with the assignment of subjective values to choice options [6].

### 3.1 An economic choice paradigm using nutrient rewards

**Fig. 1:**
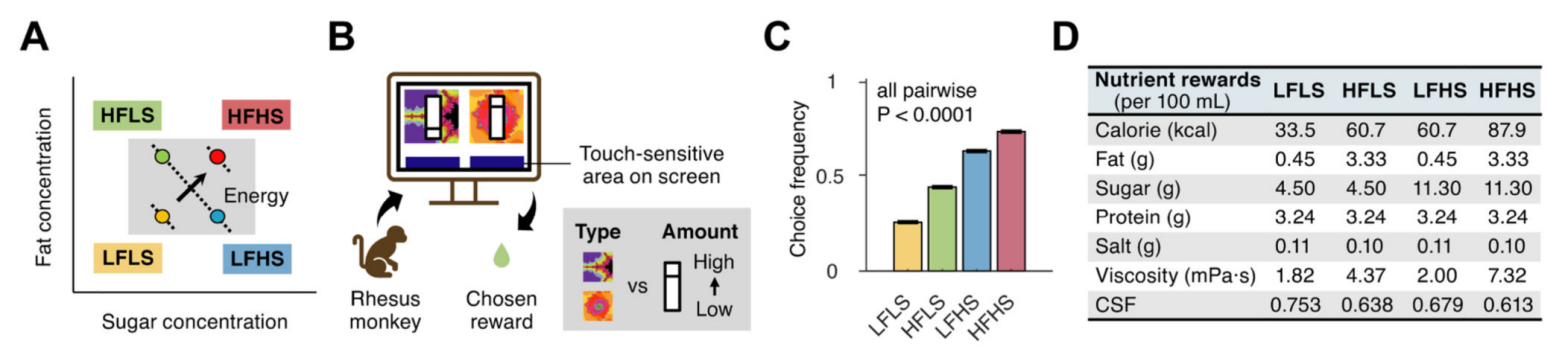
Nutrient-reward choice task in monkeys. **(A)** Factorial nutrient-reward design. LFLS: low-fat low-sugar; HFLS: high-fat low-sugar; LFHS: low-fat high-sugar; HFHS: high-fat high-sugar milkshakes. **(B)** Choice task. Monkeys choose repeatedly between two of the four food rewards, typically for 300–500 trials per testing session. Food rewards are indicated by pre-trained visual cues; reward amounts are cued by magnitude bars. **(C)** Reward preference. Choice frequencies (± SEM) for each food reward across sessions and animals (*N* = 55,205 trials). **(D)** Nutrient compositions (per 100 mL) and measured oral-texture parameters of milkshakes. Viscosity: measured by rotational rheometer at 19 °C (see Fig. 3B); CSF: coefficient of sliding friction, measured by biological tribometer using fresh pig tongues (see Fig. 3C). CSF normalized to water (CSF = 1). Adapted from [6].

To examine the influence of specific nutrients on the food choices of rhesus macaques (*Macaca mulatta*), we [6] prepared nutrient-defined liquid rewards (milkshakes) with two levels of fat and sugar contents in a factorial design (Fig. 1A): low-fat low-sugar (LFLS), high-fat low--sugar (HFLS), low-fat high-sugar (LFHS), and high-fat high-sugar (HFHS), each indicated by a pre-trained visual cue. In a computer-based choice task, the monkeys were presented with repeated binary choices between two of the four milkshake options, offered in varying amounts, and made hundreds of choices throughout each testing day (Fig. 1B). Generally, the three monkeys exhibited a stronger preference for sugar than fat: they chose the HFHS milkshake most frequently, followed by LFHS, HFLS, and LFLS (Fig. 1C). Critically, the milkshakes were carefully matched in juice flavor, temperature, and other ingredients (e.g., protein and salt) (Fig. 1D). Therefore, systematic choice biases towards specific foods could be exclusively attributed to the constituent nutrients (fat or sugar) and nutrient-related oral-sensory properties.

### 3.2 Behavioral evidence for nutrient-specific preferences

**Fig. 2:**
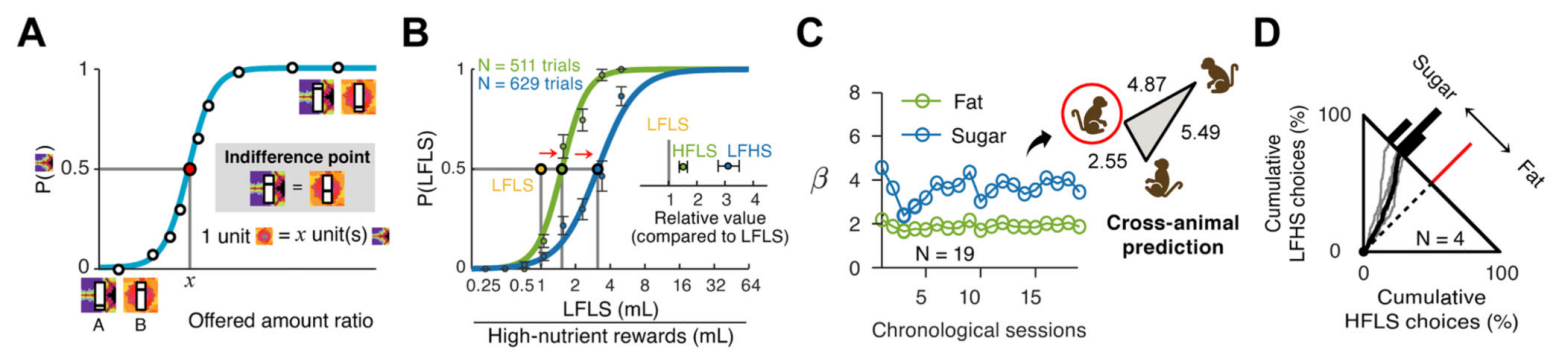
Nutrient-specific food valuation in monkeys. **(A)** Schematic of a psychometric curve and subjective-value quantification (indifference point) from monkeys’ choices between two different liquid rewards. The psychometric curve links the monkey’s choice probability for a given food (cued by different fractals) to the ratio in which the two foods were offered. The indifference point (equal choice probability) indicates the relative value of the foods. **(B)** Subjective value for high-fat (HFLS, green) and high-sugar food rewards (LFHS, blue) compared to low-nutrient reference (LFLS, yellow). Data from two testing sessions in one monkey. Inset: indifference points. Error bars: SEM. **(C)** Stable subjective nutrient values and individual differences. Left: fat (green) and sugar (blue) regression coefficients (β) for fat and sugar on choices across testing sessions in one monkey (red circle). Right: visualization of Preference Dissimilarity Index (PDI, see [15]) across three monkeys. Larger distances in the triangle indicate more dissimilar nutrient preferences. **(D)** Cumulative choices between isocaloric high-fat and high-sugar rewards indicate preferences for sugar over fat, irrespective of energy content. Gray lines: single-session result; black line: session-average choice trajectory in one monkey. Dashed line and red line: trajectory predicted using hypothetical energy-maximization strategy. Adapted from [6].

To test whether monkeys exhibit preferences for specific nutrients when making food choices, we used an economic approach that quantified individual monkeys’ subjective values for specific nutrients, based on observed systematic ‘quality-quantity’ trade-offs between nutrient-defined liquids that differed only in fat or sugar levels [6]. We used a repeated-choice procedure that mimicked the bargaining process in a market scenario: when a buyer proposes a price lower than the seller’s subjective value for a product, the seller will reject the offer. However, when the proposed price gradually increases to reach the seller’s subjective value, the seller becomes willing to sell the product and accept the deal. Analogously, in this study, monkeys started to accept the less preferred low-nutrient milkshake only when its offered quantity exceeded the subjective value for the high-nutrient milkshakes (Fig. 2A).

The monkeys preferred both fat and sugar relative to a low-nutrient reference option, indicated by ‘indifference points’ established from repeated choices. These indifference points identified the specific ratios between the offered amounts at which the monkeys were willing to forego the preferred reward (HFLS and LFHS) in exchange for a larger amount of the less preferred reward (LFLS) [6]. Indifference points provide empirical measures of how much fat and sugar are subjectively valued by the animals. For instance, in one of the monkeys, the high-fat milkshake was worth 1.5 times more than the reference milkshake, while the high-sugar milkshake (with the same calorie content) was valued 3.1 times more than the reference (Fig. 2B). Once these subjective exchange rates are identified, amounts of different reward types can be converted to a common subjective-value scale, analogous to converting prices across currencies for direct comparison. These subjective nutrient values provided a reliable metric of individuals’ nutrient preferences, demonstrated by their stability within each monkey over extended testing periods (up to two years) while also reflecting subjective, idiosyncratic preferences that differed between individuals (Fig 2C). The consistency of the monkeys’ choices with the converted subjective values suggests that these values function as ‘subjective exchange rates,’ which the animals use during food valuation and decision-making. This evidence for economic valuations of specific nutrients in monkeys—revealed quantitatively by the monkeys’ choices—complements prior experimental investigation of economic nutrient-valuations in humans [8,18,20,21,68].

### 3.3 Energy maximization does not explain nutrient preference

An influential ecological concept posits that energy serves as the common currency animals maximize when making foraging decisions [69,70]. In this framework, food options are evaluated solely based on their calorie content, largely irrespective of macronutrient compositions. However, direct behavioral evidence from economic preference tests in monkeys challenges the energy-maximization hypothesis [6]. In the study described above, monkeys exhibited a consistent preference for high-sugar rewards over high-fat rewards, even when the two options were matched in calorie content (Fig. 2D). These nutrient-specific choice biases suggest that specific nutrients, rather than total energy content alone, influence food choice. Models in normative ecology theory have been developed to explain how animals should acquire and balance multiple nutrients during foraging [44,71–73]. Studies in free-ranging primates have provided considerable evidence for nutrient-adaptive food choice [10,43,74–77], yet corresponding evidence from experimental studies in primates remains scarce. Notably, the monkeys in the study above were tested in conditions of controlled liquid intake but made food choices in the absence of nutrient challenges or deficit states. The monkeys’ fat and sugar preferences may thus differ from those of wild macaques, and resemble more closely human eating phenotypes in free-choice scenarios.

In summary, these data indicate that monkeys make choices between foods consistent with the assignment of economic values to specific nutrients. This decision-making process supports flexible trade-offs between competing food options and would enable adaptive food choices that align with changing nutritional needs.

## 4 Oral-texture properties mediate fat preferences

**Fig. 3:**
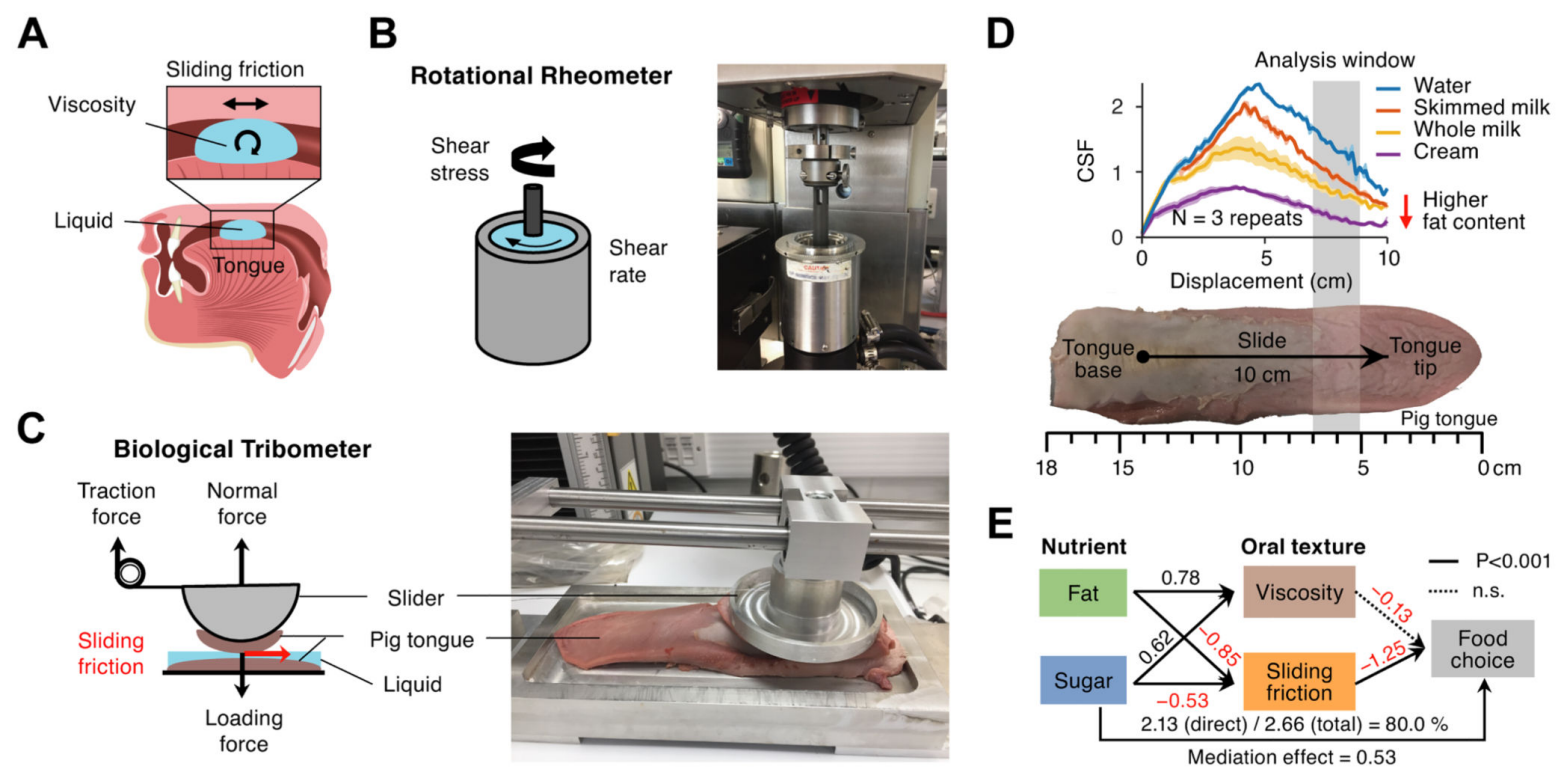
Oral texture mediates fat preference. **(A)** Food oral processing. Viscosity: resistance of liquid food to oral motion produced by molecular interactions within the food material; sliding friction: resistance to relative motion against contacting oral surfaces. **(B)** Rotational rheometer for viscosity measurement. **(C)** Biological tribometer using fresh pig tongues as biological surfaces to measure CSF under oral conditions (introduced in [6]). **(D)** Oral friction measured in the biological tribometer reflects the fat content of liquid foods, with lower CSF for high-fat foods. **(E)** Oral texture, especially CSF, mediates the influences of fat content (but not sugar content) on monkeys’ food choices. Adapted from [6].

A mechanistic question arises naturally when investigating nutrient-based food choices: What are the sensory mechanisms by which primates detect and discriminate between different nutrients? While sugar can be readily detected by oral sweet-taste receptors [78], primates seem to sense fat primarily through its distinctive mouthfeel via specialized oral mechanoreceptors [78–80], although separate gustatory mechanisms for fat-sensing exist in rodents [81]. The physical basis for somatosensory fat detection involves coalescing fat droplets that form an adhering layer on oral surfaces, thereby reducing the sliding friction of oral tissues and influencing oral mechanosensation [17,82,83]. To investigate the potential behavioral relevance of this oral somatosensory mechanism for fat-specific valuation, we focused on two key physical parameters that govern oral mechanosensation in liquid foods: viscosity and sliding friction (Fig. 3A).

### 4.1 Oral rheology and tribology measurements: viscosity and sliding friction

Viscosity and sliding friction are two interrelated yet distinct physical properties of liquids that are crucial for oral-texture perception. Viscosity refers to a liquid’s resistance to motion under an applied force, defined as the quotient of shear stress to shear rate, and is typically measured in a metal rotational rheometer (rheology) [84] (Fig. 3B). In contrast, sliding friction measures the resistance to relative motion between contacting surfaces (tribology), which varies significantly with the surface microstructure [85,86]. Therefore, conventional instruments using metal surfaces cannot accurately replicate the biological conditions during oral processing of food texture [87]. To address this limitation, we devise a custom tribometer, inspired by prior research [87], that uses fresh pig tongues to measure the coefficient of sliding friction (CSF) of testing liquids [6] (Fig. 3C). This pig-tongue tribometer simulates the mechanical conditions during oral processing by applying a constant loading force to the anterior mobile portion of the tongue and measuring the resistance as oral tissues slide over different liquid foods. In control liquids with varying fat content and in our experimental food stimuli used for behavioral experiments, this biologically relevant setup effectively distinguished liquids in terms of fat-dependent sliding friction (Fig. 3D).

### 4.2 Relating sliding friction and viscosity to behavioral food choices

Using this biologically plausible measure of sliding friction, we tested whether monkeys’ choices between foods reflected the foods’ parametrically varying degrees of oral sliding friction. We found that the monkeys’ preference for dietary fat is derived largely from a preference for oral sliding friction (Fig. 3E). Specifically, in a structural-equation modelling approach, the impact of fat on monkeys’ preference choices was fully mediated by a combination of sliding friction and viscosity; by contrast, sugar effects were only partially mediated by these oral-texture parameters. Typically, the monkeys’ preferred foods with lower sliding friction, indicating preferences for foods with a smooth, oily texture typical of high-fat stimuli. Previous studies had shown that human food ratings reflected viscosity and CSF in distinct ways [13,16] yet their impact on food preferences using economic choice tasks had not been not studied. Thus, the findings using a biologically plausible tribometer indicate that oral sliding friction provides important sensory information about the fat content of foods to guide monkeys’ food preferences [6].

## 5 A neural mechanism for fat preference in the human brain

### 5.1 Measuring neural responses to oral sliding friction in the human brain

To investigate the neural mechanisms by which oral texture influences reward valuation, we [4] used functional magnetic resonance imaging (fMRI) to measure neural responses when human volunteers orally sampled small quantities of liquid foods varying in fat and sugar content (‘milkshakes’). To control the oral-mechanical stimulation produced by the tested liquids, participants were trained to perform standardized tongue movements during oral food-sampling, followed by rinses with a control liquid (‘artificial saliva’ [88]) after each liquid delivery. Psychophysical ratings conducted both within and outside the MRI scanner confirmed that the purpose-designed milkshakes reliably produced oral sensations that reflect their nutrient and sensory properties. Additionally, participants complete a modified Becker–DeGroot–Marschak auction task to measure how much they were willing to pay for each milkshake, reporting their subjective value in single-trial responses [89].

**Fig. 4:**
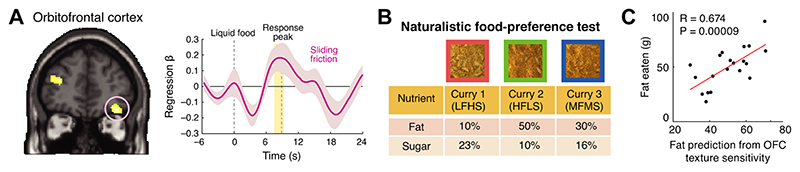
A neural mechanism in the human orbitofrontal cortex for preferring high-fat foods based on oral texture. **(A)** Activity in the human orbitofrontal cortex—measured with functional MRI—correlates with the oral sliding friction produced by fatty liquids in the mouth. Left: Neural responses to oral sliding friction during oral food processing. Pink circle: the orbitofrontal cortex (OFC). Right: temporal dynamics of the neural oral sliding-friction responses (time-resolved regression coefficient for sliding friction) in the OFC. Shaded area: SEM. **(B)** Food design for naturalistic food-preference test. Vegetarian ‘curry’ meals varied in fat and sugar content but were matched in appearance: LFHS: low-fat high-sugar; HFLS: high-fat low-sugar; MFMS: medium-fat medium-sugar. **(C)** Correlation between OFC fat-texture sensitivity (measured in the MRI scanner) and total fat consumption (g) in life-like eating test. Adapted from [4].

Previous human neuroimaging studies demonstrated that fat-containing foods activate the orbitofrontal cortex (OFC), the oral somatosensory cortex (oSSC), amygdala, hypothalamus and pregenual anterior cingulate cortex [19,22,23,25,90]. Moreover, the OFC and connected areas have been implicated in integrating sensory food properties [23,50,91–97] and other choice-related factors, such as nutrients, healthiness and tastiness [8,18,98–100]. Using texture-defined liquids and behavioral measures of economic food valuation, we found that the OFC and oSSC encode oral texture parameters (viscosity and CSF) during oral food processing (Fig. 4A) [4]. Neural activity produced by the liquid foods in the OFC also correlated with subjective ratings of oiliness, a potential subjective correlate of CSF. Notably, distinct activity patterns in OFC encoded both the foods’ oral-texture properties and the subjective values individual participants assigned to the milkshakes. These findings suggest that the human OFC plays a key role in translating specific oral-texture inputs and related sensations into subjective food valuations.

### 5.2 Human OFC activity predicts fat intake in naturalistic eating tests

To test the validity of OFC responses to fatty foods for predicting aspects of food choice in naturalistic, life-like situations, we [4] invited participants from the fMRI study mentioned above to participate in an *ad libitum* eating test (Fig. 4B). This eating test was originally used to dissociate fat and sugar preferences in obese human subjects with impaired central melanocortin signaling [101]. In this test, the subjects attended a lunch session during which they freely and repeatedly selected from three versions of a vegetarian ‘quorn korma’ curry meal presented in separate serving trays. The meals were matched in flavor and visual appearance but varied in relative fat and sugar concentration. This naturalistic test mimicked the eating experience encountered in typical dining halls; it thus provided an ecologically valid comparison to the highly controlled experimental conditions of the MRI scanner.

Relating subjects’ food choices in this test to their previously measured neural responses, we found a significant relationship between OFC activity and fat consumption in this naturalistic context. Subjects whose OFC responses were more sensitive to fat-related oral-texture qualities during oral food processing in the MRI scanner consumed more fat during the naturalistic eating test (Fig. 4C) [4]. Importantly, this neural-behavioral relationship was not found for the total amount of food consumed, indicating that high fat consumption was related to a specific preference for fat rather than high food intake irrespective of nutrient content. Moreover, the effect was specific to the OFC but was not found for a control brain area, the oSSC. By linking neural activity during controlled experiments to behavior in naturalistic eating scenarios, these findings highlight the pivotal role of the human OFC in translating oral-texture qualities into subjective food values that guide eating behavior.

## 6 Nutrient-sensitive reinforcement learning

The findings summarized above demonstrate that humans and macaques make food choices as if they assigned nutrient values to foods. Such nutrient-specific valuation would allow primates to determine how much of a given nutrient to acquire from available food options. This ability is particularly critical in natural environments, where fluctuating food and nutrient availabilities require adaptive decision-making strategies to maintain nutritional homeostasis [36,43]. A key mechanism for achieving this balance involves tracking the nutrient compositions of food items from experience [37].

**Fig. 5:**
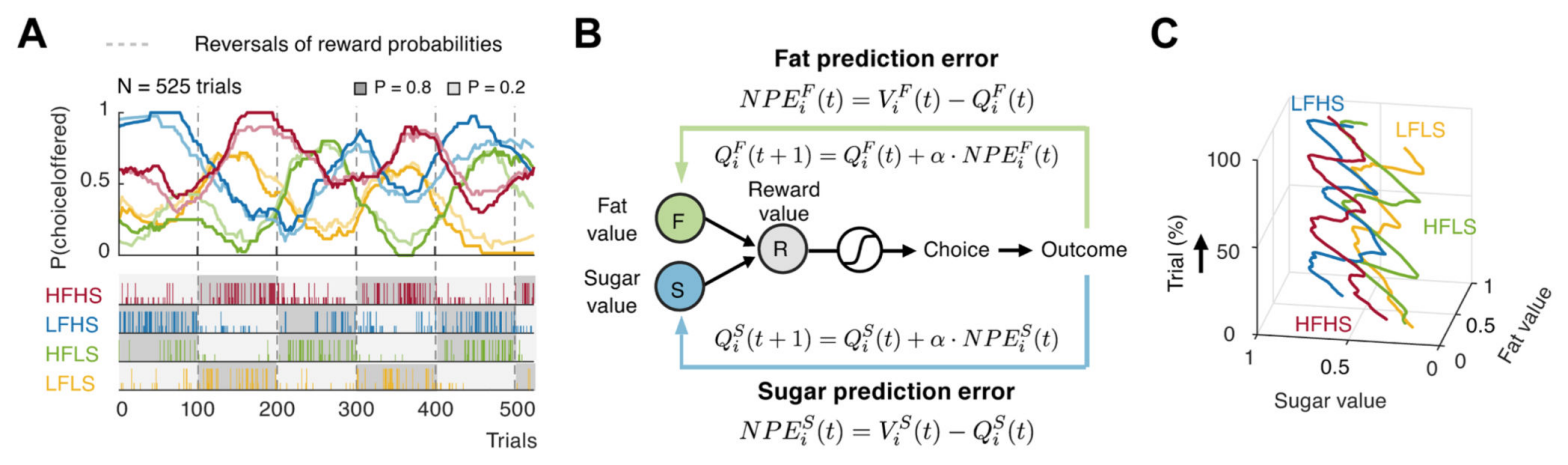
Nutrient-sensitive reinforcement learning in monkeys. **(A)** Data from one example reversal-learning session comparing the monkey’s choice probabilities with predicted choices of a nutrient-sensitive reinforcement learning model. Monkeys chose from visually cued nutrient rewards (LFLS, yellow; HFLS, green; LFHS, blue; HFHS, red) with different, varying probabilistic outcomes (dark gray blocks: *P*= 0.8 of receiving a large (vs. small) reward of the chosen food; light gray blocks: *P*= 0.2). Top: running-averaged choice probabilities. Solid lines: monkey’s choice data; faint lines: predicted choices of a nutrient-sensitive reinforcement learning model. Dashed lines indicate probability reversals (*P*= 0.8 → 0.2 or *P*= 0.2 → 0.8). Bottom: Choice and reward outcomes. Long marks: chosen and received large rewards; short marks: chosen and received small rewards. **(B)** Nutrient-sensitive RL model. Fat and sugar values for each option are integrated to a reward value and compared between options to guide choices. Experienced outcomes update values and expectations. The modelled fat and sugar value that a simulated learner assigns to a choice object *i* on trial *t* are updated from nutrient-specific prediction errors for fat (*NPE*^*F*^) and sugar (*NPE*^*S*^) separately, based on the differences between subjective experiences of received nutrients (*V*) and expected nutrient values (*Q*) on the previous trial (*t* − 1). *α* = learning rate ∈ [0, 1]. **(C)** Dynamic updating of subjective values. Same data as in (A). The trajectories show how the subjective values for the different food rewards as estimated by the RL model evolve as a function of choices and experienced rewards over the time course of a testing session.

To experimentally investigate nutrient-adaptive food choices, we presented monkeys with visual cues associated with nutrient rewards varying in fat and sugar content [37]. Unlike conventional probabilistic reversal-learning tasks that typically involve only binary choice outcomes (‘rewarded’ or ‘unrewarded’), our experimental design introduces fluctuating reward probabilities (high probability= 0.8 or low probability= 0.2) of liquid food rewards that differ in fat and sugar content (Fig 5A). This approach allowed us to examine reinforcement learning from experienced physiologically distinct nutrient rewards.

Our data revealed that the nutrient composition of rewards significantly influences learning and decision-making processes [37]. The monkeys constantly shifted their choices toward options offering higher probabilities of delivering preferred nutrients. Specifically, the monkeys’ choices for cues associated with identical reward probabilities but different nutrient rewards reflected the monkey’s subjective nutrient preference (e.g., compare yellow and red curves in Fig. 5A). These behaviors are effectively modeled by a ‘Nutrient-sensitive Reinforcement Learning’ framework, which iteratively updates the values of each option based on the subjective valuation of the received nutrient components from previous trials [37] (Fig. 5B). Thus, these findings suggest parallel RL systems each track the contributions of different nutrients that construct overall food values (Fig. 5C), enabling nutrient-specific regulation of food intake.

Recent theories suggest that multiple parallel RL systems enhance adaptability in complex, dynamic environments with conflicting goals [102]. Unlike homeostatic RL models, which explain subjective values as the motivational drives to recover from specific deprivation states [103], our findings align with theories proposing that subjective values drive long-term survival benefits [43,77]. Specifically, we argue that nutrient-specific food valuation is a general learning and decision mechanism that engages multiple parallel RL systems for different nutrients to finetune immediate nutritional status and achieve long-term evolutionary fitness. Accordingly, we predict that neurons in the key macaque brain areas for reward, reinforcement learning and decision-making detect and encode specific nutrients and nutrient-correlated sensory food qualities to support these processes.

This discussion raises the issue of whether behavioral preferences and potential neural signals related to specific nutrients genuinely reflect nutrients or related, nutrient-correlated sensory attributes (visual cue properties, flavors, oral textures). For example, a recent study showed that neurons in OFC and amygdala recorded in a decision task encode different flavors of fluid rewards cued by visual stimuli, which for OFC was shown to reflect the monkeys’ flavor preferences [104]. We suggest that addressing this important issue will require careful experimental tests that can dissociate nutrient from sensory neural coding, such as nutrient-specific satiation or appetite modulation [105], using different visual cue sets for the same nutrient rewards (to distinguish visual from nutrient coding), using artificial sweeteners and thickeners and manipulation of flavor-/texture-nutrient associations. Given the extensive multisensory (visual, taste, olfactory, oral-somatosensory) inputs of the key reward structures OFC and amygdala in primates, and their connections to the hypothalamus [106–108], we expect these neural systems to provide rich representations of both the sensory and nutrient components of food rewards to implement preferences, learning and adaptive food intake.

## 7 Nutrient balance in the geometric framework for nutrition (GFN)

**Fig. 6:**
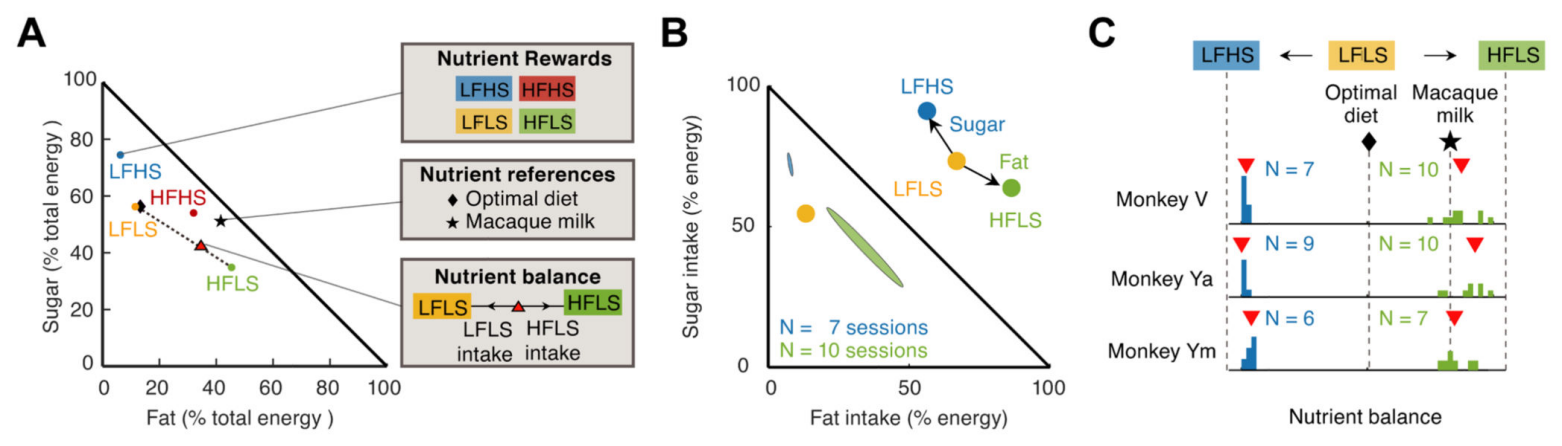
Geometric framework for nutrition (GFN). **(A)** Schematic of GFN framework [6], based on [109]. The mixture triangle visualizes (1) nutrient compositions of food options (2) nutrient reference points (3) nutrient intake balance in a common nutrient space (% total energy). **(B)** Experimental data from monkeys’ nutrient choices. Averaged nutrient-intake balance when exposed to high-fat (green, *N*= 7) or high-sugar (blue, *N*= 10) rewards. **(C)** Comparisons between dietary reference points (determined from the literature) and nutrient-intake balance from repeated food choices.

Nutrient balance, i.e., maintaining stable proportions of nutrients, is important for health and survival [36]. Accordingly, many animals adopt nutrient-balancing food-choice strategies rather than prioritizing specific, individual nutrients during foraging [16,50,85]. For example, wild macaques will adapt their intake of particular foods to seasonal variations in food availability to maintain stable nutrient balances [10, 43]. Regulation of energy-intake from different nutrient sources in primates has also been linked to seasonal thermoregulatory challenges [10]. The *Geometric Framework for Nutrition* [109–111] provides a versatile analytical tool to investigate how animals distribute their food choices across key macronutrients—fat, sugar, protein. However, it has rarely been used to explain dynamic nutrient intake in experimental repeated food choices as in our paradigm. Using this framework in macaques (Fig. 6A), we compared (1) the animals’ nutrient consumption from food choices in single testing sessions, (2) the nutrient composition of available food options, and (3) approximately optimal nutrient targets in a common ‘mixture triangle,’ based on the percentage of total energy from each macronutrient [112]. This approach quantified the monkeys’ foraging strategies in terms of the dynamically evolving nutrient balance resulting from behavioral choices in relation to dietary reference points [6].

We found that the monkeys’ aggregated food choices—reflecting their individual nutrient preferences—consistently occupied stable regions within the nutrient space (Fig. 6B). As observed in humans, the nutrient balance resulting from the monkeys’ food choices was strongly influenced by nutrient availability. When high-fat and high-sugar liquids are accessible, the monkeys strongly prefer these energy-dense food options, leading to a nutrient balance that deviates from reference points (Fig. 6C). The reference points included the recommended diet composition for adult laboratory macaques consisting of a low-fat, high-protein and intermediate-carbohydrate diet [112], and macaque milk, the exclusive nutrient source for macaque infants [113]. This behavior resembles human eating patterns when high-fat and high-sugar foods are readily available [1,114].

Complementing previous ecological studies that quantified the feeding behavior and nutrient balance in wild primates [43,75–77,110], our application of the geometric framework [109] in controlled food-choice experiments in macaques in the laboratory [6] can provide valuable additional insights into how nutrient balances evolve dynamically over repeated choices and potentially into the underlying neural mechanisms. Future work could combine this approach with experimental manipulations of baseline diet composition, nutrient-specific satiation, neurophysiological recordings and neural interventions, to advance mechanistic understanding of neural food-intake control in primates.

## 8 Conclusion

This review summarized recent experimental and conceptual advances in understanding preferences for specific nutrients and their associated sensory properties in both macaques and humans [4,6,37]. The main findings suggest that monkeys make food choices based on preferences for specific nutrients, rather than solely maximizing energy intake. Subjective values, inferred from choices and formally defined for nutrient and oral-texture food components effectively explain the observed food choices and individual differences. One key oral-texture variable, the CSF, mediates in large part both monkeys’ and humans’ preferences for high-fat foods [4,6]. The human orbitofrontal cortex responds to fat in the mouth by signaling the sliding friction produced by fatty foods on oral surfaces and integrating this oral-texture signal with subjective economic value. When reward outcomes change unexpectedly, as in natural environments, monkeys prioritize learning from rewards associated with their preferred nutrients by assigning higher values to these nutrients, suggesting ‘nutrient-sensitive reinforcement learning’ [37].

These results reveal some of the principles by which sugar, fat and related sensory food qualities influence formally defined economic food preferences and reinforcement learning. Our findings have implications for understanding the behavioral and neural mechanisms underlying human preferences for high-fat and high-sugar foods. Accordingly, the findings can help develop novel foods that replace fat and sugar with low-calorie alternatives that are palatable and rewarding. Future studies could combine this nutrient-reward paradigm with neurophysiology experiments [38] and behavioral, neural, and pharmacological interventions to uncover neural mechanisms underlying primate (including human) feeding behavior. Specifically, the reward value of protein and its neural basis in primates including humans is an area of interest given its importance in food-intake regulation [9,115]. Thus, the research program reviewed here on the behavioral and neural mechanisms for nutrient reward in macaques and humans represents a promising approach for investigating neural reward pathways for human-like feeding behavior and obesity.

## Data Availability

This is a review article; data avaialbility statements are included in the original papers reviewed in this paper.
